# Reconstructing signal during brain stimulation with Stim-BERT: a self-supervised learning model trained on millions of iEEG files

**DOI:** 10.3389/frai.2025.1502504

**Published:** 2025-02-18

**Authors:** Karthik Menon, Thomas Tcheng, Cairn Seale, David Greene, Martha Morrell, Sharanya Arcot Desai

**Affiliations:** ^1^NeuroPace Inc., Mountain View, CA, United States; ^2^Department of Neurology, Stanford University, Palo Alto, CA, United States

**Keywords:** self-supervised learning, machine learning, EEG, epilepsy, brain, big data, BERT

## Abstract

Brain stimulation has become a widely accepted treatment for neurological disorders such as epilepsy and Parkinson’s disease. These devices not only deliver therapeutic stimulation but also record brain activity, offering valuable insights into neural dynamics. However, brain recordings during stimulation are often blanked or contaminated by artifact, posing significant challenges for analyzing the acute effects of stimulation. To address these challenges, we propose a transformer-based model, Stim-BERT, trained on a large intracranial EEG (iEEG) dataset to reconstruct brain activity lost during stimulation blanking. To train the Stim-BERT model, 4,653,720 iEEG channels from 380 RNS system patients were tokenized into 3 (or 4) frequency band bins using 1 s non-overlapping windows resulting in a total vocabulary size of 1,000 (or 10,000). Stim-BERT leverages self-supervised learning with masked tokens, inspired by BERT’s success in natural language processing, and shows significant improvements over traditional interpolation methods, especially for longer blanking periods. These findings highlight the potential of transformer models for filling in missing time-series neural data, advancing neural signal processing and our efforts to understand the acute effects of brain stimulation.

## 1 Introduction

Brain stimulation has emerged as a highly effective technique for treating a range of neurological disorders, including Parkinson’s disease and epilepsy (Benabid, 2003; Fisher et al., 2010; Kringelbach et al., 2007). Devices such as the NeuroPace RNS System and the Medtronic Percept have received FDA approval and are now in clinical use (Geller et al., 2017; Nair et al., 2020; Weaver et al., 2009; Salanova et al., 2021). In addition to delivering therapeutic stimulation, some of these devices are capable of recording brain activity, which is particularly valuable for understanding the brain’s response to stimulation (Swann et al., 2018; Morrell, 2011; Sellers et al., 2021). Analyzing brain activity during and surrounding stimulation pulses can provide crucial insights into how these pulses modulate neural dynamics. For instance, studies have demonstrated that electrical stimulation can acutely reduce spectral power in brain activity immediately following stimulation (Rønborg et al., 2021; Sohal and Sun, 2011). However, a significant challenge arises from the fact that recordings are often either blanked or contaminated by stimulation artifacts during the delivery of stimulation pulses, rendering them unsuitable for direct analysis. The ability to reconstruct brain activity during these periods of artifact could therefore be invaluable for both clinical and research applications.

Previous efforts to reconstruct or denoise stimulation artifacts have primarily utilized signal processing and machine learning techniques such as template subtraction, adaptive filtering, and signal interpolation (Hines et al., 1996; Bahador et al., 2023). Each of these methods, however, has inherent limitations (Mumtaz et al., 2021). Template subtraction, for example, requires precise identification of artifact patterns, which can be difficult to achieve (Hashimoto et al., 2002). Adaptive filtering, a real-time artifact rejection technique, adjusts its coefficients continuously to better estimate and remove the artifact while preserving the neural signal. Nevertheless, obtaining an appropriate reference signal for adaptive filtering can be challenging, especially if the artifact is poorly defined or if the reference signal contains neural components. Additionally, adaptive filtering may not be effective with different types of stimulation pulses (Hua, 2020). Both template subtraction and adaptive filtering are only applicable in scenarios where the neural signal is captured during stimulation and is ineffective if the recording is completely blanked (Mumtaz et al., 2021). Interpolation methods are often employed to handle missing or blanked data, but their performance can degrade significantly when the duration of blanking is prolonged (Lepot et al., 2017). Additionally, interpolation methods only take a few samples surrounding the stimulation blanking to reconstruct the signal and hence may not capture longer-term context to reconstruct lost signal. Given these limitations, there is a need for more advanced approaches to reconstruct brain activity during periods affected by stimulation artifacts. In fact, several recent studies have demonstrated tremendous potential of deep learning techniques in advancing neural data analysis and EEG classification tasks (Waqar et al., 2019; Hussain et al., 2019; Iqbal et al., 2024). Following that, this study proposes the use of transformer-based models with attention mechanism (Vaswani, 2017; Devlin, 2018) to overcome the challenges associated with the above methods, potentially offering a novel solution for reconstructing neural signals during stimulation blanking.

Transformer-based Large Language Models (LLMs) have demonstrated remarkable capabilities in natural language processing tasks, particularly in filling in missing words within a sequence by leveraging contextual understanding (Naveed et al., 2023). A notable model is BERT (Bidirectional Encoder Representations from Transformers) which employes bidirectional self-attention mechanisms, enabling it to grasp the context of words in a sentence from both directions (Devlin, 2018; Koroteev, 2021). BERT’s architecture consists of multiple layers of transformers that process input text as tokens, and the model is pre-trained on extensive text corpora using a self-supervised learning technique known as masked language modeling (MLM) (Devlin, 2018). During MLM, certain tokens in the input sequence are randomly masked, and the model is trained to predict these masked tokens. Following pre-training, BERT can be fine-tuned on specific tasks with labeled datasets, achieving state-of-the-art results across various NLP benchmarks (Devlin, 2018; Koroteev, 2021). While BERT and similar models are traditionally designed for text, the core principles of tokenization and masked modeling can be applied to time series data, such as EEG. Recent studies utilizing self-supervised training of BERT models on EEG data to create embedding models for downstream classification tasks have shown promising performance (Wang et al., 2023; Kostas et al., 2021; Hollenstein et al., 2021). In this paper, we leverage a large intracranial EEG (iEEG) dataset, comprising over 4 million 90-s signals from 475 NeuroPace patients to train Stim-BERT specifically for reconstructing data lost during stimulation blanking. Our results show that this approach significantly outperforms interpolation techniques in reconstructing blanked data, with the model’s performance advantage increasing as the duration of blanking grows.

## 2 Methods

### 2.1 The NeuroPace RNS system and intracranial EEG (iEEG) records

The NeuroPace RNS System is an FDA-approved responsive neurostimulation device designed to detect and stimulate abnormal brain activity, specifically for the treatment of drug-resistant focal epilepsy (Morrell, 2011). The device can connect to up to two leads, which may be strip leads, depth leads, or a combination of both. To date, the system has been implanted in over 5,000 patients, resulting in the collection of more than 17 million intracranial EEG (iEEG) records.

Data for this study were obtained from the NeuroPace® RNS® System clinical trials. All study protocols were approved by the US FDA and the institutional review boards of the participating investigation sites. All participants gave written informed consent. The RNS System Feasibility, Pivotal, LTT and PAS studies are registered on CllinicalTrials.gov (NCT00079781, NCT00264810, NCT00572195, and NCT02403843, respectively).

**Figure 1:**
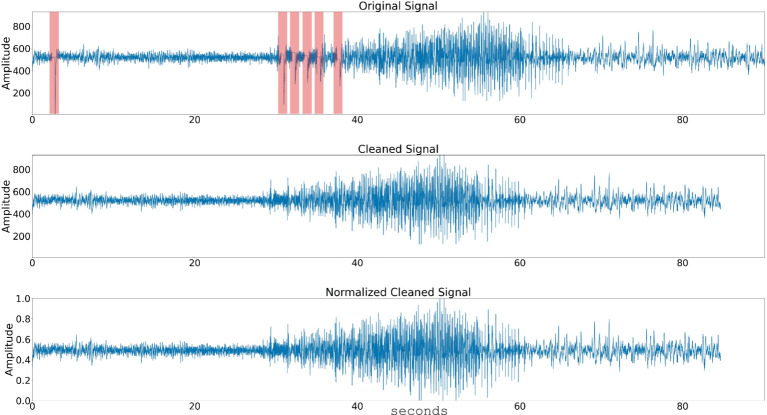
Data preprocessing steps. The raw time series signal is passed through a stimulation artifact cleaning step. The signal amplitude is normalized and the spectral power in 3 or 4 frequency bands is computed. Top panel shows a time series signal with five consecutive stimulation blanking periods (highlighted with arrow). Center panel shows the time-series signal with the stimulation blanking portions cleaned, and bottom panel shows the time-series signal’s amplitude normalized.

During neurostimulation, the recording amplifiers are temporarily blanked, leading to segments of iEEG data that are flat or blanked (Figure 1 top panel shows an example of six stimulation pulses). The device can be programmed to deliver up to five stimulation therapies when abnormal brain activity is detected. Each stimulation therapy consists of two bursts, with the duration of each burst ranging from 10 milliseconds to 5 s, typically around 100 milliseconds. Over 35% of all iEEG records captured using the NeuroPace RNS System contain at least one instance of stimulation blanking, followed by an amplifier recovery artifact, which appears as a sharp spike followed by a rapid decay lasting a few hundred milliseconds.

Each iEEG record typically includes four channels of data, with two channels recorded from each lead. The data is sampled at 250 Hz per channel (Jarosiewicz and Morrell, 2021).

### 2.2 Data pre-processing

#### 2.2.1 Data split into training, validation, and test

**Figure 2:**
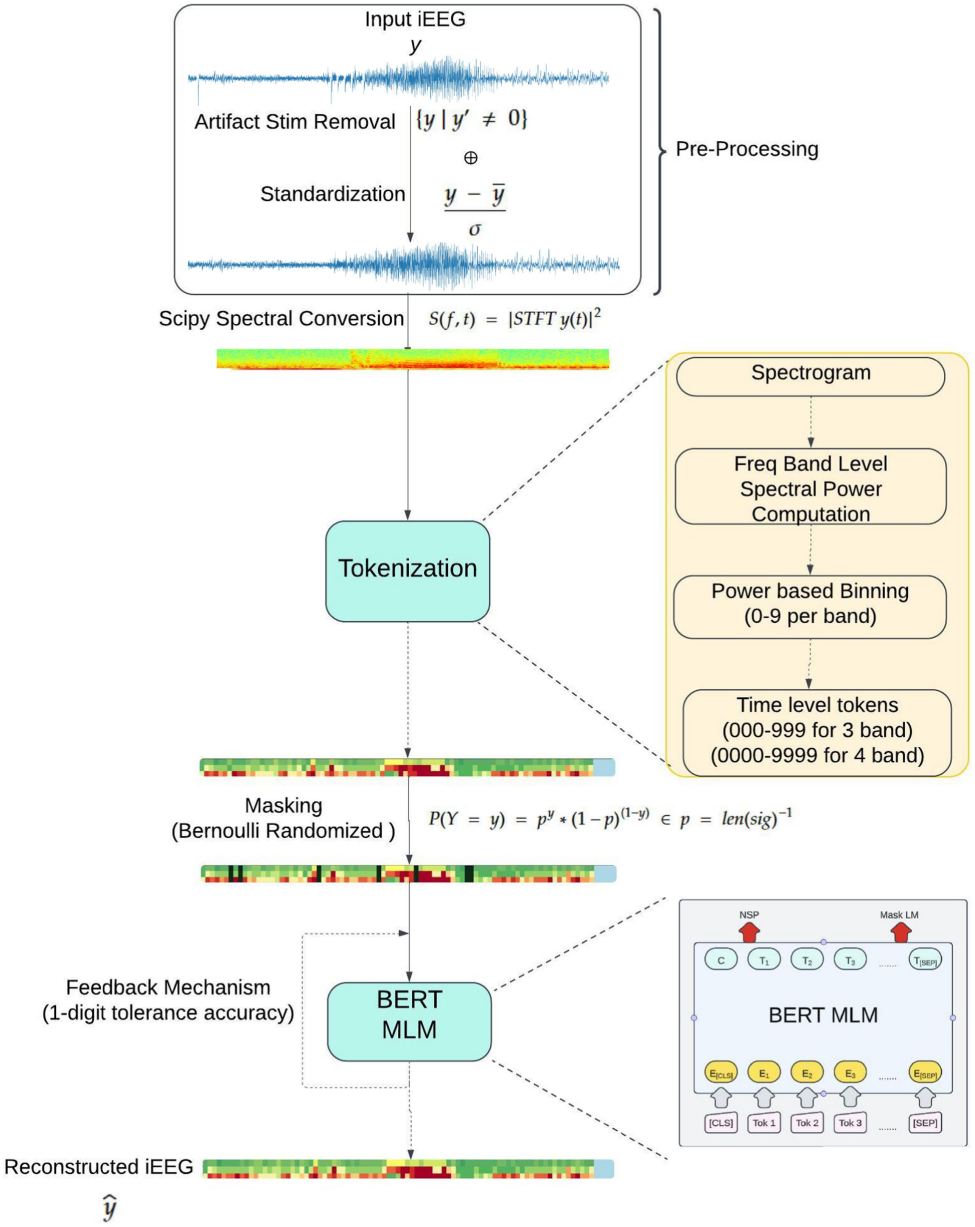
The iEEG time series signal (typically 90 s long) is passed through a stimulation artifact removal step. Spectral power in 1 s bins was computed using the scipy python package. Tokenization was performed to result in 90 tokens. A total 18% of the tokens in the training dataset were masked for training a bi-directional transformer model. The validation dataset was used to select the training hyperparameters and to determine the number of epochs of training. The test dataset was used to report the performance of the trained Stim-BERT model.

For this study, approximately 1.4 million iEEG records (each record with up to 4 channels) from 475 patients were used. The patient data was shuffled and divided into training, validation, and test sets (Table 1), ensuring no overlap of iEEG records from the same patients across these data groups. The training dataset was used to train the Stim-BERT model, as described in a later section. An overview of the iEEG processing and Stim-BERT model training steps are shows in Figure 2. The validation dataset was employed to determine the optimal number of training epochs and hyperparameters, while the test dataset was used to evaluate the model’s performance. All data used in this study were obtained from RNS patients enrolled in clinical trials, with consent provided for research purposes.

**Table 1:** The number of iEEG channels in the train, validation, and test datasets is summarized in the table.

Dataset	Train	Validation	Test
Number of patients	380	48	47
Number of iEEG channels	4,653,720	527,950	472,265

#### 2.2.2 Stimulation artifact rejection, spectral power extraction

Stimulation artifact were identified and removed using previously published methods (Desai et al., 2019; Sun et al., 2018; Barry et al., 2021). Specifically, the first derivative of the signal was computed to detect flat portions caused by stimulation blanking. An additional 125 samples following the blanking period were excluded to account for amplifier recovery artifact. The time series segments before and after these periods were then concatenated, effectively eliminating the artifacts. This approach successfully removed all blanking artifacts and most of the amplifier recovery artifacts (Figure 1).

After artifact removal, the signal was normalized by calculating the z-score using the formula:


$$\mathrm{Signal}=\left(\mathrm{Signal}–\mathrm{mean of signal}\right)/\mathrm{standard deviation of the signal}$$

This normalization adjusted the signal amplitude.

The spectral power of the signal was computed using SciPy with a window size of 250 samples, focusing on three frequency ranges: 0–13 Hz (encompassing delta, theta, and alpha power), 13–35 Hz (beta power), and 35–125 Hz (gamma power) (Desai et al., 2019). Since the sampling rate was 250 Hz, this window size produced three power values (low, medium, and high frequency bands) per second of data. For example, a 90-s time series would yield a 90×3 spectral power matrix.

A similar approach was applied using four frequency bands instead of three: 0–8 Hz (delta and theta), 8–13 Hz (alpha), 13–35 Hz (beta), and 35–125 Hz (gamma). The rationale for splitting the lower frequency range for the 4 frequency band case was to achieve higher resolution in the lower frequency range, considering the 1/f nature of neural activity (Lombardi et al., 2017).

### 2.3 Tokenization

The next step involved converting the analog spectral power values in the 3 (or 4) frequency bands into a fixed number of quantized bins. For each frequency band, a bin range of 0–9 (10 bins) was used. In the three-band scenario, this resulted in 1,000 possible combinations of bins (range: 000–999), where a value of 000 would indicate very low spectral power across all three bands, and 999 would indicate very high spectral power. In the four-band case, this method resulted in 10,000 possible combinations of bins (range: 0000–9999). The rationale for selecting 3 (or 4) frequency ranges was to keep the number of tokens limited to a few thousand, aligning with the vocabulary size used for training large language models (LLMs) like BERT, which has a vocabulary size of 30,522 tokens created using WordPiece tokenization (Devlin, 2018).

**Figure 3:**
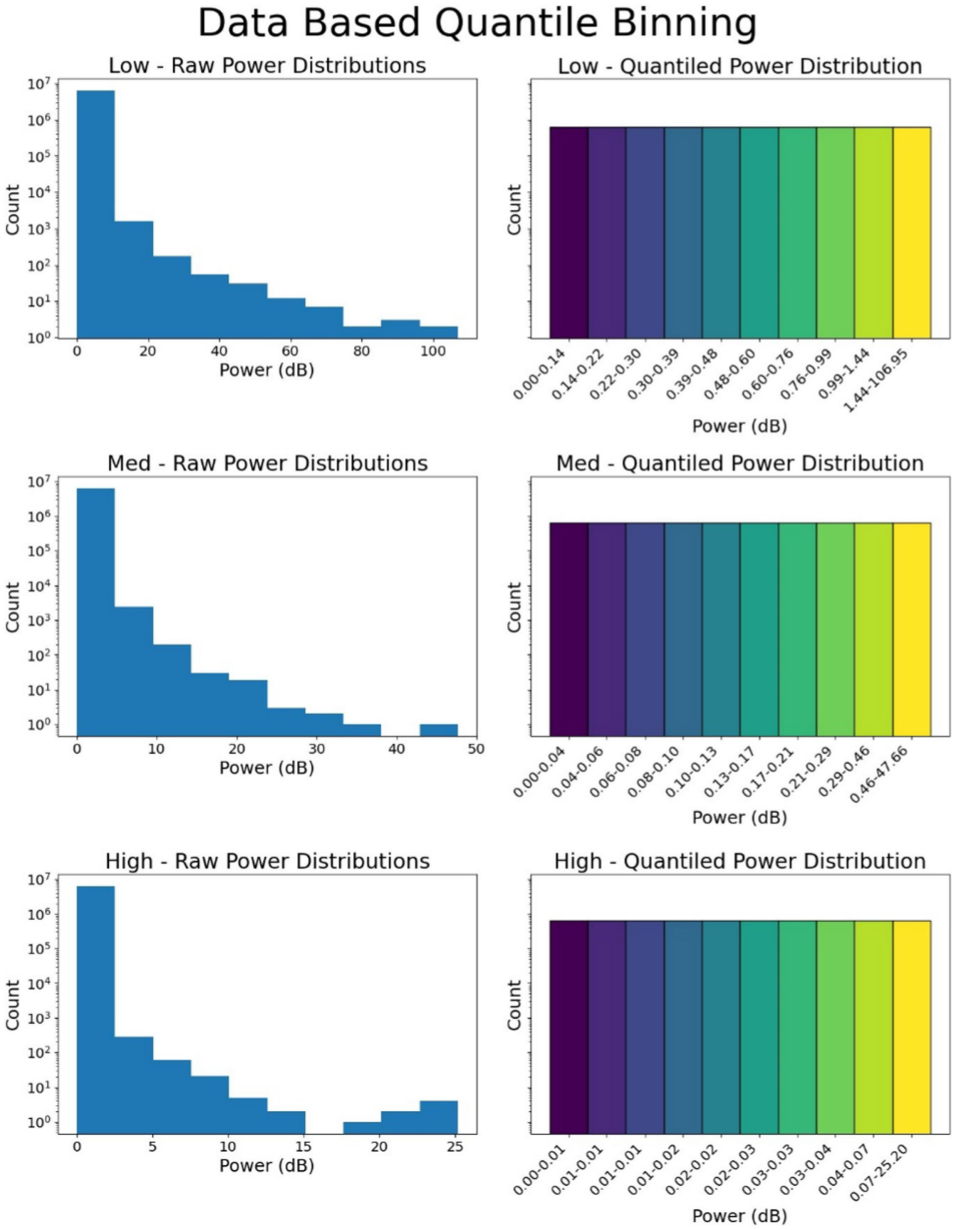
Binning of data and quantization. The spectral power in 3 or 4 frequency bands is quantized into bins. In this figure, quantization of 3 frequency bins is shown. Top panel shows power distribution and dB quantization boundaries for the low frequency range (0–13 Hz), the middle panel and bottom panels show the power distribution and dB quantization for 13–35 Hz (medium frequency), and 35–125 Hz (high frequency).

The spectral power in each frequency range was not uniformly divided into 10 bins due to the unequal distribution of data, with higher spectral power having fewer samples, reflecting a long-tail distribution. To address this, 250,000 iEEG records (~500 per patient) were randomly selected from the training dataset. The spectral power in each frequency range across these records was divided into quantile-based bins using pd.qcut, ensuring even distribution across 10 bins for each frequency range. Once the bin boundaries were determined, all iEEG records in the training, validation, and testing datasets were converted into these spectral bands and quantized into bins 0–9 for each frequency band (Figure 3).

Most iEEG records captured by the NeuroPace RNS System are 90 s long, resulting in 90 tokens per channel of iEEG data after tokenization. For iEEG records shorter than 90 tokens, token 0 was used to fill in the missing data toward the end of the record. For records longer than 90 s, only the first 90 s of the record were used and the remaining tokens were discarded. Using this method, all iEEG records were converted into 90 tokens for each channel of iEEG.

### 2.4 Random masking of tokens and Stim-BERT model training

**Figure 4:**
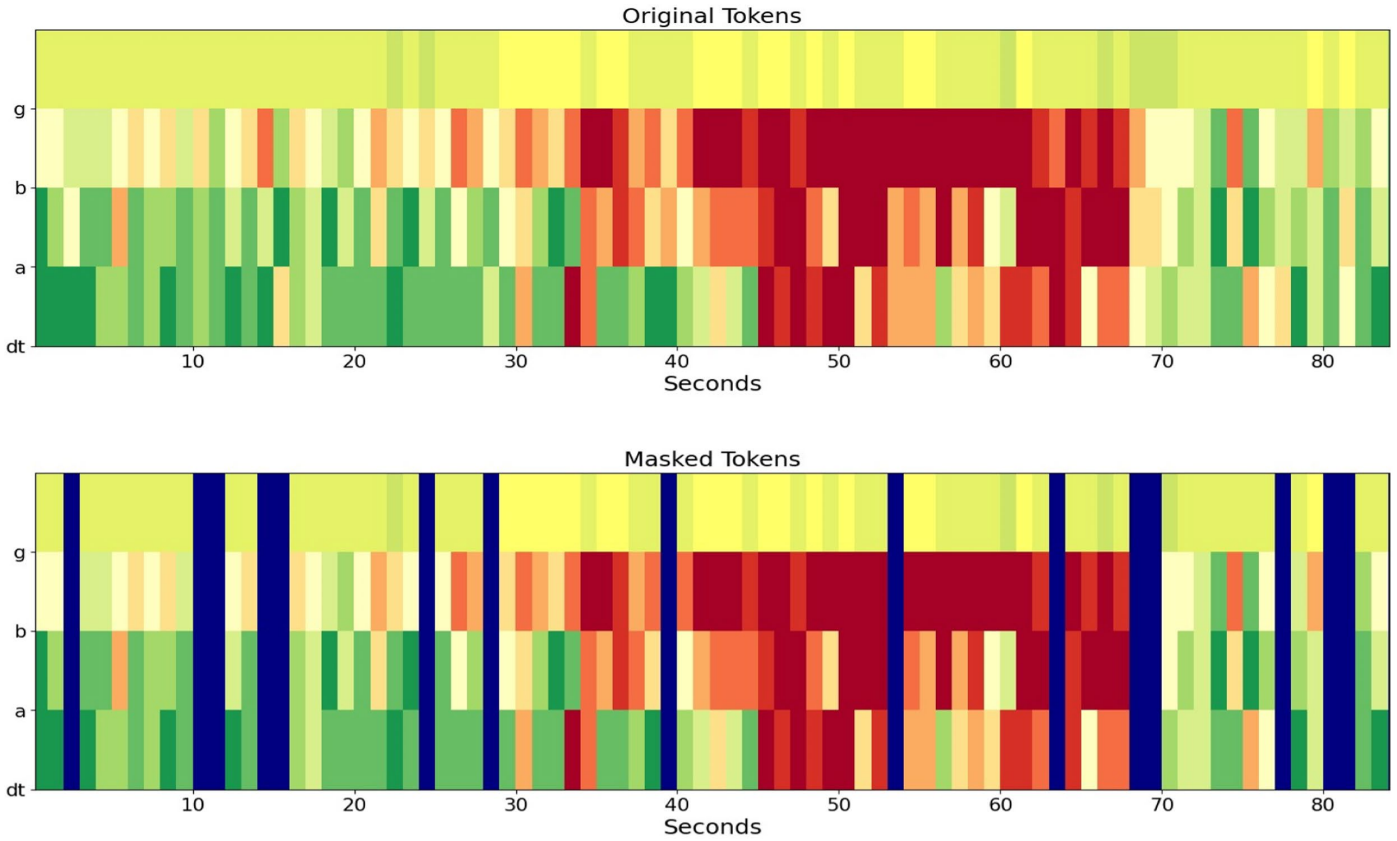
Example of original and masked tokens for the 4-frequency band case. On the y-axis, dt is delta and theta band, a is alpha, b is beta, and g is gamma band.

During Stim-BERT model training process, 18% of the tokens (16 out of 90 tokens) within each iEEG channel were masked, using a token value of 1,000 to indicate masking for both the three-band and four-band cases. The training objective was to predict these masked tokens, with the cross-entropy loss function defined accordingly for optimization (Figure 4).

The model’s performance on the validation dataset was used to fine-tune the hyperparameters. The final hyperparameters used in this study are as follows:

- VOCAB_SIZE: 1001 (10,001 for four-band)
- MODEL_DIMENSION: 496 (dimension of the model’s hidden state)
- MAX_LEN: 90 (corresponding to 90 tokens)
- MASK_PROBABILITY: 0.18 (adjusted from the default 0.15 to account for the presence of zero tokens at the end)
- NUM_TRAINING_EPOCHS: 30 (determined through trial and error)
- NUM_HIDDEN_LAYERS: 6
- NUM_ATTENTION_HEADS: 8
- OPTIMIZER: Adam with a learning rate of 1e-6
- EARLY_STOP_PATIENCE: 10

### 2.5 Model evaluation and performance on test dataset

To evaluate the model’s performance, a tolerance of ±1 digit was applied to each digit within a token. For the three-band case, if the actual token is “456” and the model predicts “345,” it is considered correct. However, a prediction of “256” would be incorrect. The same principle applies to the four-band case, where each digit in the 4-digit token also has a tolerance of ±1. This approach allows small deviations in predictions to be considered accurate, providing a more robust measure of model performance.

Overall performance is measured as the mean accuracy percentage, determined by dividing the total number of correctly predicted tokens (within the tolerance) by the total number of tokens in each 90 s iEEG file and multiplying that ratio by 100. This yields an accuracy metric for each iEEG channel. The aggregate performance across all test iEEG channels is then calculated using the mean and standard error of the mean.

The test dataset involved masking between 1 and 10 tokens, using two different masking approaches. In the first approach, individual tokens were arbitrarily masked within the 90-token sequence. In the second approach, 1–10 consecutive tokens were masked at an arbitrary position within the sequence to assess the model’s ability to reconstruct data when up to 10 s of signal are lost due to stimulation artifacts. All masked tokens used for model evaluation were confined to the actual signal length within the 90-s sequence. For example, if the iEEG record only produced 80 tokens (with the record being 80 s long), masked tokens were restricted to the first 80 tokens. Masking beyond this range would have artificially inflated Stim-BERT’s reconstruction accuracy.

### 2.6 Comparison with other methods

The performance of the reconstructed tokens generated by Stim-BERT model are compared with a simple Interpolation method. In this Interpolation approach, the context size was varied from 1 token on either side of the missing token up to 5 tokens. The predicted token value was calculated by averaging the tokens on either side of the missing token(s) and using that average as the estimated value.

Accuracy of reconstruction of Stim-BERT is compared against Interpolation and random token methods and t-test is used to test for significance.

## 3 Results

### 3.1 Stim-BERT training and validation

Training of Stim-BERT stopped after 21 epochs for the three-band case and 20 epochs for the four-band case with an *early_stop_patience* of 10 epochs. In both scenarios, there was a notable decrease in both training and validation loss throughout the training process. For the three-band case, the training loss initially measured 6.9, ultimately decreasing to 0.702 after 21 epochs, with a corresponding validation loss of 0.720. Similarly, for the four-band case, the training loss started at 9.282 and concluded at 0.987 after 20 epochs, with a validation loss of 1.105 at the 20th epoch.

### 3.2 Performance of Stim-BERT on test dataset and comparison with other methods

**Figure 5:**
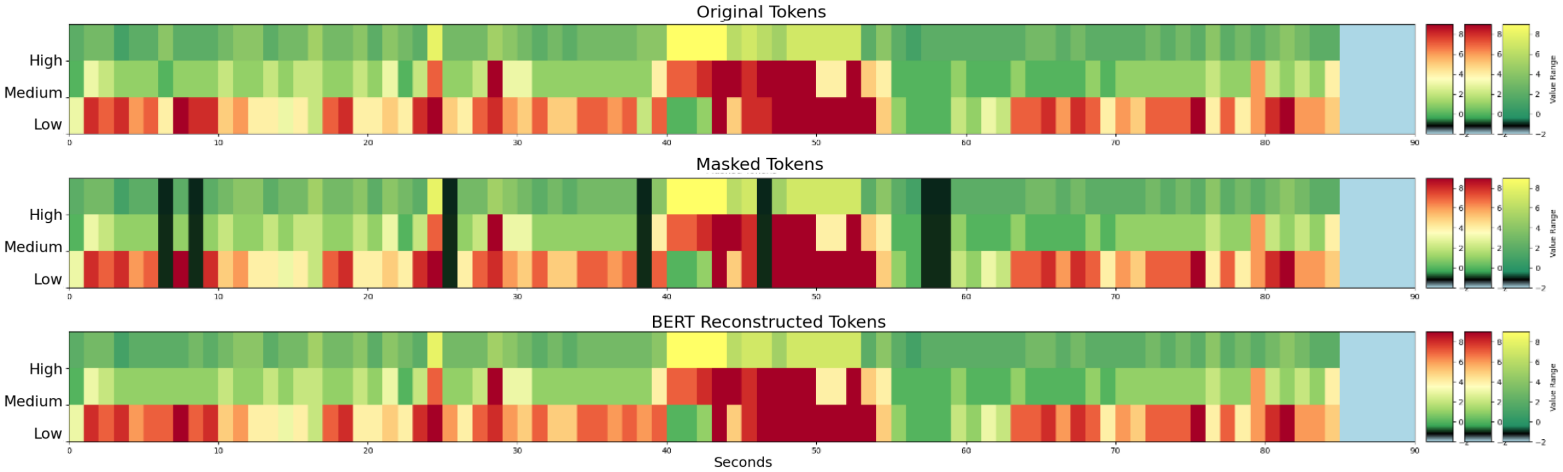
(Top) Example 3 band tokens for a 90 s iEEG file. (Middle) Arbitrarily selected individual tokens masked. (Bottom) Reconstruction of masked tokens with Stim-BERT. On the y-axis, low is delta, theta, and alpha bands, medium is beta band, and high is gamma band.

**Figure 6:**
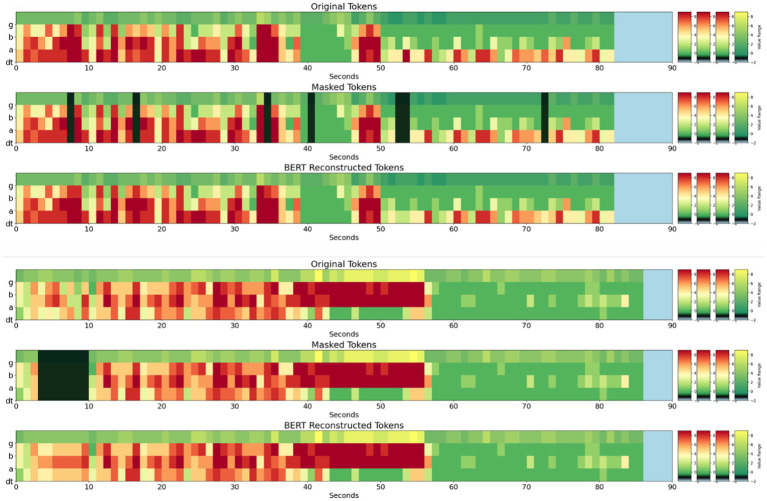
(Top panel – 3 subplots) Original, masked, and Stim-BERT reconstructed tokens for individual masked 4-frequency band tokens. (Bottom Panel—3 subplots) Original, Masked, and Stim-BERT reconstructed tokens for consecutively masked 4-frequency band tokens. On the y-axis, dt is delta and theta bands, a is alpha band, b is beta band, and g is gamma band.

Stim-BERT achieved a masked token reconstruction accuracy of approximately 30.5% when 1 to 10 individual tokens were masked at random locations within a test iEEG channel of 90 tokens (Figures 5, 6). In contrast, the Interpolation method’s accuracy was about 25.1%, significantly underperforming compared to the Stim-BERT reconstruction approach. The random token method, however, yielded an accuracy of about 2.2%, which was substantially lower than both the Stim-BERT and Interpolation methods. All three methods showed similar accuracy when masking between 1 and 10 tokens arbitrarily (Tables 2, 3) i.e., Stim-BERT based reconstruction accuracy was somewhat steady around 30% when 1–10 tokens were arbitrarily masked. Similarly, Interpolation based reconstruction accuracy was around 25%, and random token method reconstruction accuracy was around 2% when 1–10 tokens were arbitrarily masked.

**Table 2:** Performance of Stim-BERT and other methods on arbitrarily masked individual tokens (top) and arbitrarily masked consecutive tokens with three frequency bands.

Number of individual tokens masked	Stim-BERT		Interpolation		Random	
	Mean	SEM	Mean	SEM	Mean	SEM
1	30.662	0.127	25.13	0.119	2.235	0.041
2	30.91	0.094	25.238	0.088	2.243	0.029
3	30.904	0.08	25.202	0.075	2.217	0.023
4	30.765	0.072	25.108	0.068	2.225	0.02
5	30.838	0.067	25.171	0.064	2.254	0.018
6	30.808	0.063	25.172	0.06	2.24	0.017
7	30.644	0.06	25.222	0.057	2.244	0.015
8	30.523	0.058	25.123	0.055	2.223	0.014
9	30.508	0.056	25.125	0.053	2.228	0.014
10	30.508	0.054	25.253	0.052	2.236	0.013
Number of consecutive tokens masked						
1	30.701	0.142	25.216	0.133	2.251	0.046
2	30.571	0.108	24.309	0.102	2.232	0.032
3	30.333	0.093	23.646	0.089	2.223	0.026
4	30.104	0.085	23.171	0.081	2.208	0.023
5	29.819	0.079	23.012	0.076	2.254	0.02
6	29.439	0.075	22.609	0.073	2.231	0.019
7	29.312	0.073	22.283	0.071	2.263	0.017
8	29.009	0.07	21.951	0.067	2.232	0.016
9	28.914	0.068	21.739	0.066	2.227	0.015
10	28.652	0.067	21.402	0.065	2.25	0.015

**Table 3:** Performance of Stim-BERT and other methods on arbitrarily masked individual tokens (top) and arbitrarily masked consecutive tokens with four frequency bands.

Number of individual tokens masked	Stim-BERT		Interpolation		Random	
	Mean	SEM	Mean	SEM	Mean	SEM
1	18.281	0.119	14.347	0.108	0.604	0.024
2	18.034	0.088	14.336	0.081	0.637	0.017
3	18.079	0.076	14.286	0.069	0.63	0.014
4	18.082	0.07	14.198	0.063	0.637	0.012
5	17.912	0.065	14.286	0.059	0.634	0.011
6	17.904	0.063	14.323	0.057	0.625	0.01
7	17.923	0.06	14.258	0.054	0.63	0.009
8	17.956	0.058	14.339	0.053	0.632	0.009
9	17.914	0.057	14.363	0.052	0.627	0.008
10	17.758	0.056	14.208	0.05	0.634	0.008
Number of consecutive tokens masked						
1	18.216	0.119	14.312	0.108	0.674	0.025
2	17.888	0.09	13.844	0.083	0.624	0.017
3	17.765	0.079	13.543	0.073	0.617	0.014
4	17.531	0.073	13.203	0.067	0.638	0.012
5	17.323	0.068	12.902	0.063	0.62	0.011
6	17.252	0.066	12.671	0.06	0.637	0.01
7	17.091	0.064	12.64	0.059	0.642	0.009
8	16.865	0.061	12.358	0.057	0.628	0.008
9	16.927	0.061	12.345	0.056	0.634	0.008
10	16.789	0.06	12.131	0.055	0.621	0.008

For consecutive token masking, the Stim-BERT model’s reconstruction accuracy began at 30.7% with 1 masked token. As the number of consecutively masked tokens increased, the accuracy declined by about 2%, reaching 28.7% with 10 masked tokens. In contrast, the Interpolation method experienced a more pronounced decline, with accuracy dropping from 25.1% with 1 masked token to 21.4% with 10 masked tokens, a decrease of 3.7%. The random tokenization method maintained a consistent accuracy of around 2.2% across 1–10 consecutive masked tokens (Tables 2, 3).

Similar trends were seen with the 4-frequency band case with the Stim-BERT reconstruction accuracy significantly outperforming Interpolation and random tokenization methods (Table 3).

### 3.3 Performance of Stim-BERT vs. interpolation on varying context length

Performance of the Stim-BERT model was compared to the Interpolation method, where the context length for Interpolation was varied between 1 and 5 tokens on either side of the masked token. As the context length for Interpolation increased, the reconstruction accuracy slightly improved from 22.2 to 23.8%. However, this accuracy remained significantly lower than Stim-BERT’s, which ranged from 29.3 to 28.7% (Table 4).

**Table 4:** Performance of Stim-BERT and other methods on arbitrarily masked individual tokens.

Context window for interpolation	Stim-BERT		Interpolation		Random	
	Mean	SEM	Mean	SEM	Mean	SEM
1	29.312	0.073	22.283	0.071	2.244	0.018
2	28.801	0.073	23.431	0.071	2.211	0.018
3	28.82	0.074	23.818	0.072	2.233	0.018
4	28.797	0.073	23.937	0.071	2.217	0.018
5	28.714	0.073	23.812	0.071	2.239	0.018

The context length for the interpolation method is increased up to five tokens on either side of the masked token.

### 3.4 Stimulation artifact reconstruction

**Figure 7:**
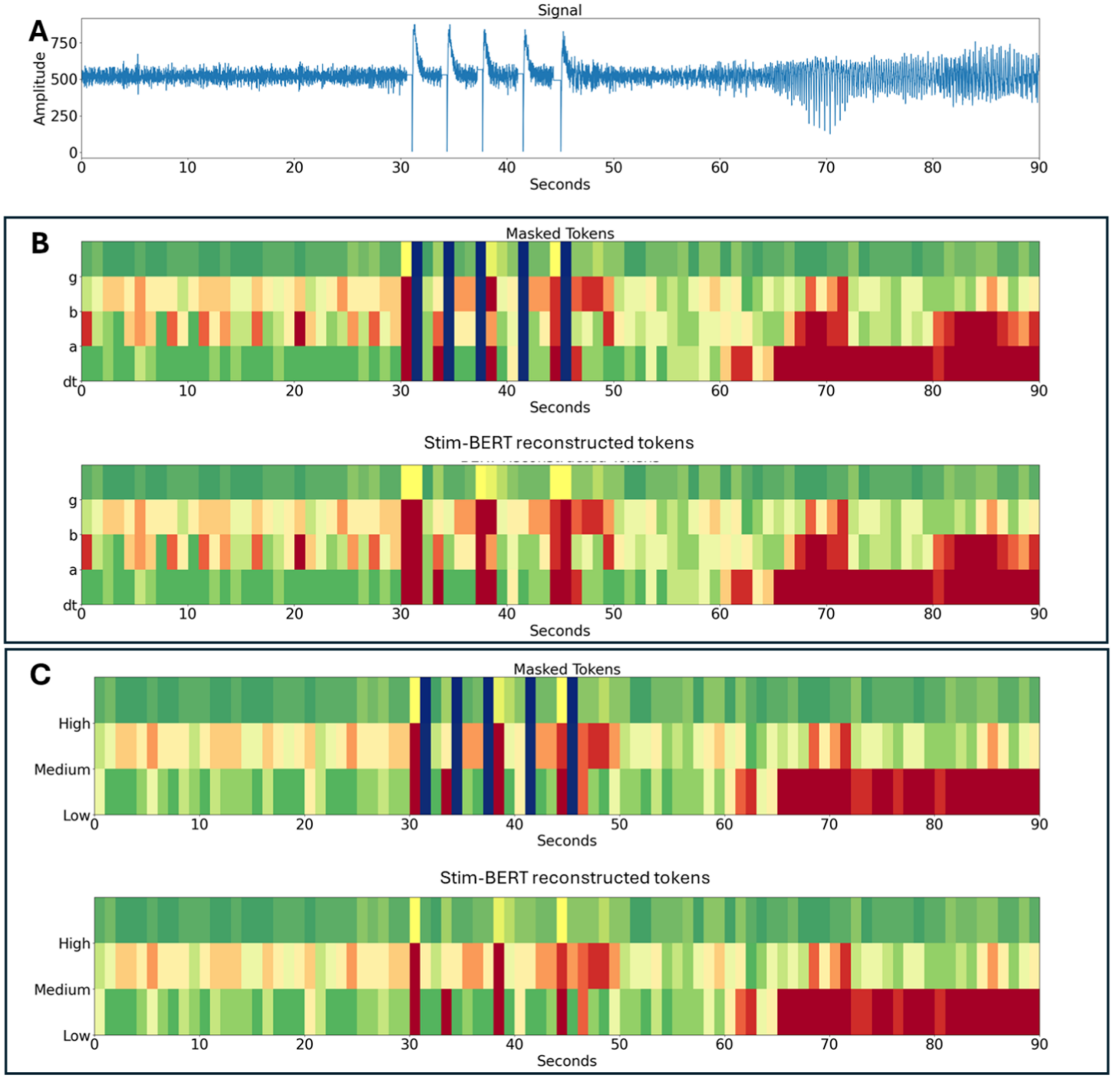
**(A)** Time series iEEG with stimulation artifact. **(B)** Time series signals are tokenized with 3 frequency bands, tokens with stimulation artifact are masked, and the activity is reconstructed with Stim-BERT. **(C)** Time series signals are tokenized with 4 frequency bands, tokens with stimulation artifact are masked, and the activity is reconstructed with Stim-BERT. On the y-axis, low is delta, theta, and alpha bands, medium is beta band, and high is gamma band.

Figure 7 illustrates an example of a time-series RNS System signal, along with the tokenized 3 and 4 frequency bands and the reconstructed signal. Although there is no ground truth available to validate the accuracy of the reconstruction in this case, the figure demonstrates the proof of concept. In this application, five consecutive stimulation artifacts can be seen in the Figure 7A. In the corresponding tokenized spectrogram (Figure 7B top), 5 masked (blue vertical lines) tokens are seen, one for each stimulation blanking period in panel A. The trained Stim-BERT model reconstructs the tokens lost to stimulation artifact (Figure 7B bottom). Similar reconstruction of the original time-series signals masked tokens can be seen with the 4-frequency band case in Figure 7C.

## 4 Discussion

Brain stimulation artifact recovery remains a major challenge in neural data analysis, with various hardware and software methods proposed to mitigate their effects (Mumtaz et al., 2021). In this study, we investigated the potential of using a transformer-based model to reconstruct data lost due to brain stimulation artifact, employing self-supervised learning on a large intracranial EEG (iEEG) dataset.

The iEEG signals used in this study were sourced from patients implanted with the NeuroPace RNS System (Morrell, 2011; Jarosiewicz and Morrell, 2021). To process the time-series data, spectral power in 3 and 4 frequency bands was extracted from non-overlapping 1-s windows and quantized into a few thousand tokens. The token count was constrained to a similar order of magnitude as the vocabulary size in language models (Devlin, 2018). For the three-band case, this approach resulted in 1,000 tokens, and for the four-band case, 10,000 tokens. The three-band reconstruction case achieved an accuracy of approximately 30%, while the four-band reconstruction case resulted in an accuracy of about 18% using Stim-BERT. The higher accuracy in the three-band case is expected, as the model only needs to predict 3 power bands (range: 000–999, with each digit representing a quantized power band) within a ± 1 digit tolerance. In contrast, the four-band case requires predicting power across 4 bands (range: 0000–9999), with the same ±1 digit tolerance, which presents a more complex challenge. Despite the lower accuracy, the increased number of bands offers higher resolution, potentially enabling more impactful applications.

Given the widespread use of spectral data in neural analysis, it was both logical and practical to extract spectral power features from 1-s non-overlapping windows (Desai et al., 2019; Sun et al., 2018; Skarpaas et al., 2018). For higher resolution, smaller window sizes of 0.25–0.5 s can be utilized. Alternatively, a technique akin to BrainBERT (Wang et al., 2023), which varies resolution across frequency bands, could be employed. For instance, if specific frequency bands like the beta band (13–35 Hz) associated with Parkinson’s disease are of interest (Little and Brown, 2014), focusing on 3–4 sub-bands within this range could enhance reconstruction accuracy. Depending on the application, other EEG features such as spike rate (Desai et al., 2019) or phase-amplitude coupling (Edakawa et al., 2016) may also be extracted for tokenization.

Neural data inherently contains both short-term and long-term context, as evidenced by seizure prediction studies (Kuhlmann et al., 2018; Mormann et al., 2007; Acharya et al., 2018), where changes in neural signals are observed several seconds to minutes before a seizure. Therefore, a model encoding long-term context is likely more effective at reconstructing missing data during stimulation blanking than methods considering only shorter time scales. To test this hypothesis, Stim-BERT’s reconstruction accuracy was compared with Interpolation, where the context length for Interpolation was extended up to five tokens on each side of the missing token. In all scenarios, the iEEG-BERT model significantly outperformed Interpolation, with a greater margin of improvement for longer windows of continuously masked data.

In this study, a maximum context length of 90 s was used. While this context length may be sufficient for stimulation artifact recovery, other applications may benefit from longer context lengths. For instance, wearable devices that capture physiological data, such as heart rate, electrocardiogram (ECG), and other biosensor data over extended periods could utilize a longer context length model for reconstructing missing data due to intermittent device charging (Picard et al., 2001). In such applications, larger transformer-based models offering longer context lengths could be applicable to these applications (Naveed et al., 2023; Thirunavukarasu et al., 2023). A recent study by Google used self-supervised learning to train a model with 100 million parameters by masking portions of multimodel wearable data in 5 h chunks collected using Fitbit watches (Narayanswamy et al., 2024).

Due to the lack of ground truth data for stimulation artifact-masked data, the Stim-BERT model was largely trained using background EEG data with masked tokens. However, stimulation might alter the underlying iEEG signals, meaning the data reconstructed using this approach may not accurately reflect the true data during stimulation artifacts. This limitation is not unique to the methods described in this paper; it is a common challenge for all techniques attempting to reconstruct data based on context during stimulation artifacts.

The concept of using BERT-like models for neural data reconstruction has been previously demonstrated with BRAINBERT (Wang et al., 2023), which was trained for EEG representation learning. In that study, short-time Fourier transform (STFT) and Superlet methods were used to compute the spectrogram of EEG data, with entire frequency and time bands masked during model pre-training. The representations learned by the model were then passed through a linear classification layer to classify the neural data, outperforming benchmark models and demonstrating the potential of transformer-based architectures for neural data. The data were segmented into 5-s blocks. Given that stimulation artifacts can last longer than 5 s, longer data segments were necessary, hence the datasets in the current study were kept at 90 s. Additionally, only temporal masking was performed, as our model was specifically trained to fill in missing temporal data.

Although Stim-BERT was not explicitly trained to reconstruct continuously masked tokens, which would result from long stimulation bursts, it was able to predict the tokens with only a slight performance degradation compared to random masking of tokens. As the duration of continuous masking increased, the Stim-BERT model continued to outperform Interpolation methods, with a larger margin as mask duration increased. Future models specifically trained to predict continuously missing tokens are expected to further enhance performance in this area.

In summary, this study showed that: (1) time-series brain recordings can be effectively tokenized using spectral information, yielding a vocabulary size of 1,000–10,000, comparable to the vocabulary size of the BERT language model; and (2) a bi-directional transformer-based model, Stim-BERT, which captures both short- and long-term context in input data, successfully recovered neural spectral data lost during brain stimulation and significantly outperformed interpolation methods.

## Data Availability

Data supporting the findings of this study may be available upon reasonable request to the corresponding author.
